# Cell cycle progression by the repression of primary cilia formation in proliferating cells

**DOI:** 10.1007/s00018-013-1302-8

**Published:** 2013-03-09

**Authors:** Hidemasa Goto, Akihito Inoko, Masaki Inagaki

**Affiliations:** 1 Division of Biochemistry, Aichi Cancer Center Research Institute, 1-1 Kanokoden, Chikusa-ku, Nagoya, 464-8681 Japan; 2 Department of Cellular Oncology, Graduate School of Medicine, Nagoya University, 65 Tsurumai-cho, Showa-ku, Nagoya, 466-8550 Japan

**Keywords:** Cilium, Cell cycle, G0/G1–S transition, Aurora-A, HEF1, Trichoplein

## Abstract

In most cell types, primary cilia protrude from the cell surface and act as major hubs for cell signaling, cell differentiation, and cell polarity. With the exception of some cells ciliated during cell proliferation, most cells begin to disassemble their primary cilia at cell cycle re-entry. Although the role of primary cilia disassembly on cell cycle progression is still under debate, recent data have emerged to support the idea that primary cilia exert influence on cell cycle progression. In this review, we emphasize a non-mitotic role of Aurora-A not only in the ciliary resorption at cell cycle re-entry but also in continuous suppression of cilia regeneration during cell proliferation. We also summarize recent new findings indicating that forced induction/suppression of primary cilia can affect cell cycle progression, in particular the transition from G0/G1 to S phase. In addition, we speculate how (de)ciliation affects cell cycle progression.

## Introduction

The cell cycle accurately duplicates large amounts of DNA in chromosomes during the S phase and then segregates duplicated chromosomes precisely into two genetically identical daughter cells during the M phase (mitosis). Most cell cycles have gap phases, G1 and G2 (G1 → S → G2 → M). Proliferating cells monitor the process to ensure that cellular preparations are complete and DNA/chromosome conditions are suitable from G1 to S (the G1/S checkpoint), during DNA replication (the intra-S phase checkpoint), from G2 to mitosis (the G2/M checkpoint) [1–12], from the metaphase to anaphase in mitosis (the spindle assembly checkpoint) [13, 14], or at the end of cytokinesis (the abscission checkpoint) [15]. Especially during the G1 phase, cells also monitor external conditions and extracellular signals from other cells. If these environments are unfavorable for cell proliferation, cells delay their cell cycle progression through the G1 phase and can remain stable for a long time (even years) before resuming proliferation. Some scientists distinguish this resting state (known as the G0 phase) as different from the (proliferating) G1 phase. Like the G1/S transition [16–18], the G0/G1 transition (cell cycle re-entry) is suppressed by the product of the *retinoblastoma* tumor suppressor gene, pRb [19, 20]. Cyclin and cyclin-dependent kinase (Cdk) complex are also critical to promote the exit from cellular quiescence through pRb phosphorylation [19, 21, 22]. However, the mechanisms governing the establishment/maintenance of the stationary (G0 or G1) phase and cell cycle re-entry (the G0/G1 transition) are not fully understood.

On the surface of many types of quiescent cells, the elder (mother) centriole frequently nucleates the growth of a non-motile, microtubule-rich surface projection called a primary cilium [23]. Primary cilia are considered to function as chemosensors and/or mechanosensors and are implicated in several developmental signaling pathways such as the Sonic Hedgehog (Shh) and Wingless/Int (Wnt) pathways [24–30]. Dysfunction of a primary cilium is associated with a broad spectrum of diseases such as polydactyly, cranio-facial abnormalities, brain malformation, situs inversus (defects of left–right patterning), obesity, diabetes, and polycystic kidney disease (PKD) [26, 27]. The mechanisms of primary cilia formation have been discussed in other excellent reviews [26–33]. In many cells, primary cilia start to disassemble as cells re-enter the cell cycle [31, 34, 35]. There seems to be an inverse relationship between ciliation and cell cycle progression. However, there are some exceptional examples of cells that retain cilia during cell proliferation [30, 31, 35–38]. For example, many ciliated protozoans maintain their cortical cilia throughout cell division [38]. Recently, Riparbelli and colleagues [39] also reported that spermatocytes in *Drosophila melanogaster* possess cilia during two meiotic divisions. Therefore, whether a primary cilium negatively controls cell cycle progression has been a topic of discussion for a long time.

Recent studies have indicated that Aurora-A, originally identified as one of the mitotic kinases [40–43], negatively regulates ciliary dynamics in proliferating cells [44–47]. Aurora-A activity outside mitosis is required for at least two different categories of ciliary dynamics, the deciliation at cell cycle re-entry (the G0/G1 transition) [44–46] and continuous inhibition of primary cilia regeneration during cell proliferation [47]. Several recent publications have also demonstrated that forced ciliary formation/absorption can influence cell cycle progression especially at the G0/G1–S transition [47–49]. In this review, we focus on the above recent advances connecting primary cilia and the cell cycle, and discuss possible crosstalk with cell cycle regulators.

## Inhibition of primary cilia assembly/regeneration by Aurora-A

Aurora-A [also known as serine/threonine kinase-6 (STK-6); encoded by *AURKA*] was originally discovered in a screen for *D. melanogaster* mutations affecting the poles of the mitotic spindle [50]. Aurora-A localizes to centrosomes and mitotic spindles and drives multi-aspects of mitotic functions including mitotic entry, centrosome maturation, centrosome separation, and bipolar spindle formation [40–43]. Several binding proteins are known to regulate the localization, activation, and/or substrate preference of Aurora-A [42, 43].

Even in the interphase, several proteins were reported to bind and activate Aurora-A (Table 1). Aurora-A activators outside mitosis are required for at least two different categories of ciliary dynamics in proliferating cells. One is ciliary resorption when quiescent ciliated cells resume proliferation. The other is continuous suppression of aberrant cilia regeneration in proliferating cells. The members of the former category contain calcium–calmodulin (Ca^2+^/CaM; discussed in a later section) [46], human enhancer of filamentation 1 (HEF1; also known as NEDD9 or Cas-L) [44], and Pitchfork (Pifo) [45], whereas trichoplein belongs to the latter category [47] (Table 1).

**Table 1:** Aurora-A-binding proteins associated with primary cilia kinetics

Protein name	Cell cycle or stimuli	Effects on Aurora-A	Function	Ref.
Calcium–calmodulin (Ca^2+^/CaM)	Cell cycle re-entry	Activation^a^	Primary cilia disassembly	[46, 96]
	Calcium ionophores		Primary cilia disassembly	[46, 96]
	Mitosis		Some mitotic functions of Aurora-A	[46, 96]
HEF1	Cell cycle re-entry	Activation^a^	Primary cilia disassembly	[44]
	Mitosis		Some mitotic functions of Aurora-A	[44, 52]
Pitchfork (Pifo)	Cell cycle re-entry	Activation^a^	Primary cilia disassembly	[45]
	Mitosis?		Some mitotic functions of Aurora-A?	[45]
Trichoplein	Cell proliferation (especially at G1 phase)	Activation^a^	Suppression of aberrant primary cilia formation	[47]

^a^Induction of Aurora-A autophosphorylation at Thr288

**Fig. 1:**
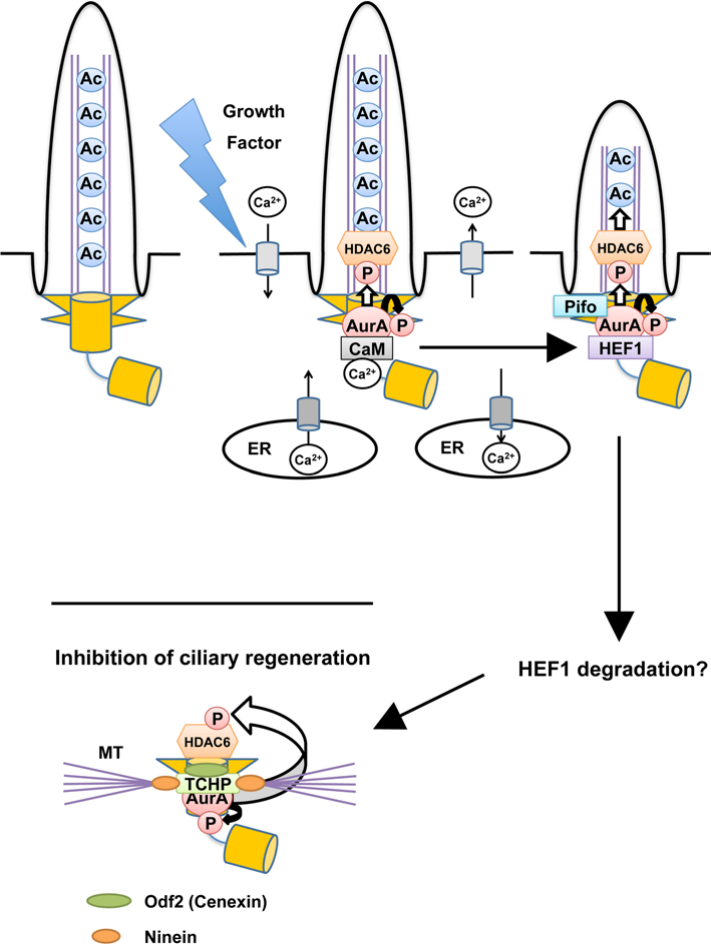
Aurora-A-mediated deciliation at cell-cycle re-entry and inhibition of ciliary regeneration in proliferating cells. At quiescent state (G0 phase), a basal body (to which a mother centriole is converted) is anchored at plasma membrane around ciliary pockets through its distal appendages [112]. Growth factor stimulation triggers calcium influx from extracellular space and/or endoplasmic reticulum (ER) to the cytoplasm. Calcium–calmodulin (Ca^2+^/CaM) binds and activates Aurora-A (through its autophosphorylation at Thr288) [96]. This activation is considered to be transient (within 3 min), but Ca^2+^/CaM also enhanced the binding between Aurora-A (AurA) and HEF1 [46], which in turn activates Aurora-A (AurA) [44]. Pifo is assumed to function in a way similar to HEF1 [45]. Aurora-A (AurA) phosphorylates and activates HDAC6, which in turn removes acetylated group on axonemal α-tubulin [44]. This deacetylation may shorten the length of axonemes. After HEF1 levels decrease [44] (likely Pifo too), trichoplein (TCHP) works as an Aurora-A (AurA) activator at the mother centriole and prevents ciliary regeneration in proliferating cells [47]. The disturbance of this process induces the G0/G1–S arrest [47]. In addition, trichoplein (TCHP) functions as a protein scaffold between Odf2 (Cenexin) and ninein; these three molecules participate in microtubule (MT) anchoring at the subdistal appendages on a mother centriole [55]

A non-mitotic function of Aurora-A was first suggested by the study of Snell’s group revealing that CALK, a distant orthologue of Aurora-A in *Chlamydomonas reinhardtii*, controls the resorption of the flagellum, an organelle similar to mammalian cilium, during mating or in response to ionic stresses [51]. Golemis and colleagues [44] observed the increase in Aurora-A-Thr288 phosphorylation (which implies Aurora-A activation [40–43]) at the basal body just after serum-deprived cultured cells were stimulated by growth factor. The treatment with Aurora-A inhibitors or siRNAs impaired ciliary disassembly after cell cycle re-entry (the G0/G1 transition), whereas the microinjection of pre-activated Aurora-A in ciliated cells accelerated ciliary disassembly [44]. Aurora-A activation in this resorption process requires HEF1 [44] (Fig. 1), a protein which the authors’ group previously identified as a novel Aurora-A binding protein [52].

In a separate study, Lickert and colleagues [45] identified Pifo as a protein expressed in the mouse embryonic node and found that it to be accumulated in the basal body at an early stage of cilia disassembly. In mice, *Pifo* haploinsufficiency led to developmental defects associated with ciliary abnormalities, such as a left–right asymmetry defect [45]. In humans, the authors also found a heterozygous R80K *Pifo* mutation in diseases related to ciliopathy [45]. Interestingly, Pifo was able to activate Aurora-A, whereas overexpression of its R80K mutant inhibited the catalytic activity of Aurora-A [45]. These observations suggest that Pifo participates in the early stage of ciliary absorption together with Aurora-A [45] (Fig. 1). This function of Pifo appears to resemble that of HEF1. Since HEF1 knockout mice have only limited defects [53], these observations lead to speculation that Pifo may work mainly during embryonic development, whereas HEF1 may function largely after the development.

The protein level of HEF1 appears to increase in response to the stimulation of serum-deprived cultured cells by growth factors but decrease by 4 h after the stimulation [44]. How does ciliary reassembly remain suppressed at subsequent cell cycle phases in proliferating cells? Our study provides important clues [47]. We found that trichoplein [a protein originally identified as a keratin intermediate filament (IF) scaffold protein [54]; encoded by *TCHP*] bound and then activated Aurora-A in vitro and the two proteins colocalized at the centrioles of proliferating cells especially in the G1 phase [47] (Table 1). Both trichoplein and Aurora-A were required for continuous suppression of ciliary reassembly, which in turn promoted proper cell cycle progression [47]. Primary cilia formation in quiescent RPE1 (h-TERT-immortalized retinal pigment epithelia) cells was also impaired by the expression of truncated mutants of trichoplein that could localize to centrioles and activate Aurora-A but not by that of the mutants lacking either ability [47]. Thus, trichoplein inhibits ciliary reassembly by activating Aurora-A [47], a mechanism similar to the HEF1 role in cilia resorption [44] (Fig. 1). All these observations suggest that Aurora-A activation in interphase requires several different Aurora-A-associated proteins, the function of which may be strictly regulated in a temporal fashion (Fig. 1).

## Cytoskeletal functions of Aurora-A-associated proteins

**Fig. 2:**
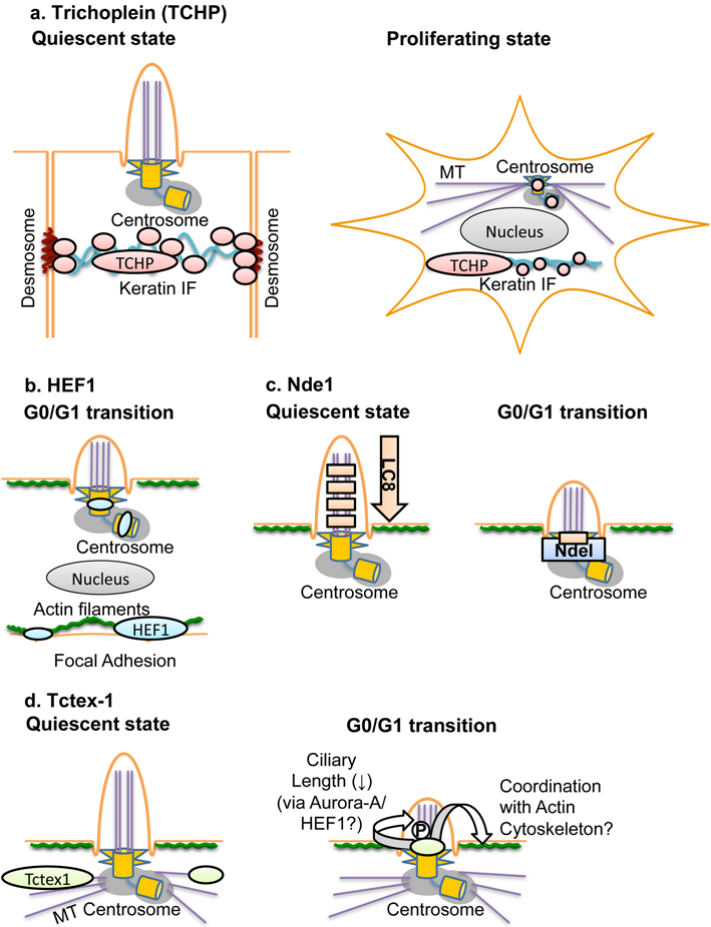
Localization and function of trichoplein (TCHP; **a**), HEF1 (**b**), Nde1 (**c**), and Tctex-1 (**d**). **a** In well-differentiated (non-dividing) epithelia, trichoplein (TCHP) is localized on keratin IFs and desmosomes [54]. On the other hand, trichoplein (TCHP) is concentrated at the subdistal to medial zone of both mother and daughter centrioles in (both epithelial and non-epithelial) dividing cells [47, 55]. Trichoplein (TCHP) functions as a protein scaffold not only to activate centriolar Aurora-A in G1 phase [47] but also to promote microtubule (MT) anchoring at the subdistal appendages of a mother centriole [55] (also see Fig. 1). **b** The protein level of HEF1 is elevated at the G0/G1 transition [44]. The precise localization in the centrosome is largely unknown, but HEF1 works as an Aurora-A activator at the G0/G1 transition [44] (also see Fig. 1). Since Aurora-A is localized at medial zone of both mother and daughter centrioles in G1 phase [47], HEF1 is assumed to be localized at least at similar areas of centrioles. HEF1 is also localized at focal adhesions, where it functions as a protein scaffold for integrin-mediated signaling including the establishment of cell attachments and migration [57, 58]. **c** The dynein light chain LC8 associates with retrograde IFT components [113], which maintain ciliary assembly [28]. Nde1 is expressed after the G0/G1 transition and localized at a basal body [48]. Nde1 recruits LC8 to the basal body through their association [48]. Sequestration of LC8 leads to the inhibition of ciliary assembly [48]. **d** In response to growth factor stimulation, Tctex-1 is phosphorylated at Thr94 and then recruited to ciliary transition zone [49]. This recruitment of phosphorylated Tctex-1 induces ciliary disassembly likely through the rearrangement of actin cytoskeleton and the activation of Aurora-A by HEF1 [49]

With regard to intracellular localization both in quiescent and proliferating cells, trichoplein is a well-studied molecule (Fig. 2a). In well-differentiated (non-dividing) epithelia, trichoplein is localized on keratin IFs and desmosomes; this localization depends on the binding to keratin proteins [54]. On the other hand, trichoplein is concentrated at centrioles in (both epithelial and non-epithelial) dividing cells and has a key role in microtubule-anchoring activity at centrosomes during proliferation [55]. This activity depends on direct interaction of trichoplein with two other centriolar proteins, Odf2 (also called Cenexin) and ninein [55]. In addition to this activity, trichoplein serves as a scaffold for centriole-associated Aurora-A, which suppresses aberrant primary cilia formation in proliferating cells [47]. Thus, trichoplein appears to translocate from keratin IFs and desmosomes to centrioles and change its function to prepare for cell proliferation at cell cycle re-entry (the G0/G1 transition).

Several studies have also illustrated critical roles for the actin cytoskeletal network in assembling primary cilia (reviewed in detail in [32]). The formation of a primary cilium in quiescent cells depends on the relationship of cellular position between nucleus and centrosome, which is largely determined by cell shape and contractility [56]. Interestingly, HEF1, originally identified as a member of a group of scaffolding proteins that includes p130Cas and Efs/Sin, also localizes to focal adhesions and act as an intermediate in a variety of integrin-dependent signaling processes, including the establishment of cell attachments and migration [57, 58] (Fig. 2b). These observations suggest the possibility that HEF1 and trichoplein may function as molecular scaffolds to synchronize cytoskeletal changes in the centrosome and cell attachments.

## Signaling pathways downstream of Aurora-A

With regard to the pathology of PKD, Golemis and colleagues [59] found that Aurora-A was overexpressed and hyperactivated in early renal cysts associated with PKD. Aurora-A bound and phosphorylated polycystin (PC) 2, which limited its calcium channel activity [59] (Table 2). PC2 (encoded by *PKD2*) dimerizes with a transmembrane receptor protein PC1 (encoded by *PKD1*) in the primary cilium and their mutations induce renal cyst formation associated with PKD [60, 61] (reviewed in detail in [62–64]). Since the cilia serve as mechanosensors in the renal tubules and flow-induced ciliary bending results in a transient increase in intracellular calcium [65], Aurora-A overactivation may play an important role in the pathological condition of PKD through PC2 inhibition.

**Table 2:** Putative Aurora-A substrates outside mitosis

Putative substrate	Phosphorylation site	Functional change	Ref.
HDAC6	Not identified	Elevation of tubulin deacetylase activity	[44]
Polycystin 2 (PC2)	Ser829	Inhibition of calcium channel activity	[59]
p53	Ser215	Abrogation of DNA binding and transactivation activity	[85]
	Ser315	Mdm2-mediated destabilization	[84]

With regard to substrates downstream of Aurora-A in ciliary resorption, Golemis and colleagues [44] first identified the tubulin deacetylase HDAC6, the activity of which was stimulated by Aurora-A-induced phosphorylation (Table 2). Ciliary resorption at cell cycle re-entry was disturbed by the suppression of HDAC6 activity through treatment with tubacin (the small molecule inhibitor of HDAC6) or HDAC-6-specific siRNAs [44]. HDAC6 activated by Aurora-A removes acetylated group on axonemal α-tubulin. Since functional analyses of αTAT1 (α-tubulin acetyltransferase 1; also known as MEC-17) demonstrated that ciliary assembly decreased in speed by strongly decreased acetylation on α-tubulin [66, 67] (reviewed in detail in [68]), deacetylation on axonemal α-tubulin may induce ciliary disassembly. Thus, the activation pathway from Aurora-A to HDAC6 most likely explains not only ciliary disassembly at cell cycle re-entry (mediated by HEF1 and/or Pifo), but also the suppression of ciliary reassembly in proliferating cells (mediated by trichoplein; Fig. 1).

Although α-tubulin acetylation is highly enriched on the axoneme, some studies have argued against the role of α-tubulin acetylation in the assembly or function of primary cilia (reviewed in detail in [32, 36, 68]). For example, cilia were morphologically normal in *Tetrahymena thermophile* where the acetylation of microtubules was strongly reduced by the expression of a non-acetylated mutant of α-tubulin [69]. The change in acetylation status of tubulin did not influence the resorption of the flagellum in *C. reinhardtii* [70], which was likely to share the Aurora-A-mediated resorption system [51]. HDAC6 knockout mice showed hyperacetylated tubulin in most tissues, but did not reveal apparent phenotypes that would be expected to result from aberrant ciliary formation [71]. Most recently, two reports provided surprising observations that revised our classic view of α-tubulin acetylation at Lys40 (a major acetylation site) as a marker of stable tubulin; the absence of this modification only reduced microtubule diameter and length in touch receptor neurons of *Caenorhabditis elegans* [72, 73]. Thus, Aurora-A may also phosphorylate other important substrates to disassemble primary cilia or to inhibit its regeneration. All of the known tubulin posttranslational modifications (PTMs) except for acetylation occur on C-terminal tails of tubulin exposed on the outer surface of microtubules, whereas the Lys40 acetylation site is located on the inside of microtubule polymers [68]. Since C-terminal tails serve as key interaction sites for microtubules-associated proteins (MAPs) and motor proteins [68], other PTMs such as glutamylation may contribute to ciliary formation rather than acetylation. Therefore, Aurora-A may phosphorylate key enzymes for other PTMs or indirectly regulate their activity.

## Effects of primary cilia dynamics on cell cycle progression

With the exception of some cells possessing primary cilia during cell proliferation, cells begin to retract their primary cilia at the G0/G1 transition (cell cycle re-entry). This relationship implies that ciliogenesis and cell proliferation may be mutually exclusive processes, but it remains controversial whether or not (de)ciliation affects cell cycle progression [30, 31, 35–37]. However, several recent publications provide some clues [47–49, 74].

Tsiokas and colleagues [48] reported Nde1, an *Aspergillums* NudE (nuclear distribution gene E; Ref. [75]) homolog 1, as a novel protein localized to the mother centriole. The protein level of Nde1 was high in mitosis but low in G0/G1 phase [48], which showed an inverse correlation with the existence of primary cilia. RNAi-mediated Nde1 depletion induced not only abnormally long cilia but also a delay in cell cycle re-entry in NIH3T3 or RPE1 cells [48] (Table 3). Nde1 shortened ciliary length via the interaction of Nde1 with a dynein light-chain protein, LC8 [48] (Fig. 2c). The authors observed a similar effect of Nde1 on ciliary length in zebra fish embryos, where Nde1 depletion reduced proliferating cells of the Kupffer’s vesicle (an organ analogous to the embryonic node), which resulted in defects of left–right patterning [48]. The cell cycle delay in Nde1-depleted RPE1 cells reverted by co-depletion of IFT88 (intraflagellar transport protein 88, also known as Polaris) or IFT20 [48] (Table 3). Since these IFT proteins are composed of IFT complex B, which contributes to anterograde transport and is essential for the assembly/maintenance of cilia and flagella [28], these results indicate that forced ciliary absorption can influence cell cycle progression [48]. The authors also demonstrated that the G0/G1–S transition can be delayed by forced induction of longer cilia through other treatments, such as the ectopic expression of a constitutively active variant of Rab8a and a brief disruption of the actin cytoskeleton by cytochalasin D [48] (Table 3).

**Table 3:** Effects of each treatment on primary cilia and cell cycle progression

Treatments	Ciliation		Cell cycle	Cultured cells	Ref.
	Length	Percentage			
Trichoplein KD		↑	G0/G1 arrest	RPE1	[47]
	(Non-ciliated cell line)		Only marginal effects	HeLa	[47]
Trichoplein KD + trichoplein OE		→	Only marginal effects	RPE1	[47]
IFT88 KD		↓	Only marginal effects	NIH3T3, RPE1	[47]
IFT20 KD		↓	Only marginal effects	NIH3T3, RPE1	[47]
Trichoplein KD + IFT88 KD		↓	Only marginal effects	RPE1	^a^
Trichoplein KD + IFT20 KD		↓	Only marginal effects	RPE1	[47]
Aurora-A KD		↑	G0/G1 arrest	RPE1	[47]
	(Non-ciliated cell line)		Mitotic failure	HeLa	[47]
Aurora-A KD + Aurora-A OE		→	Only marginal effects	RPE1	[47]
Aurora-A KD + IFT88 KD		↓	Only marginal effects	RPE1	^a^
Aurora-A KD + IFT20 KD		↓	Only marginal effects	RPE1	[47]
Chloral hydrate		↓	Only marginal effects	RPE1	^a^
Trichoplein KD + chloral hydrate		↓	Only marginal effects	RPE1	^a^
Aurora-A KD + chloral hydrate		↓	Only marginal effects	RPE1	^a^
Nde1 KD	↑		Delay in G0/G1 transition	NIH3T3, RPE1	[48]
IFT88 KD	↓		Only marginal effects	NIH3T3, RPE1	[48]
IFT20 KD	↓		Only marginal effects	NIH3T3, RPE1	[48]
Nde1 KD + IFT88 KD	↓		Only marginal effects	NIH3T3, RPE1	[48]
Nde1 KD + IFT20 KD	↓		Only marginal effects	NIH3T3, RPE1	[48]
Cytochalasin D	↑		G0/G1–S arrest	RPE1	[48]
Cytochalasin D + IFT20 KD	↓		Only marginal effects	RPE1	[48]
Rab8a Q67L expression	↑		Delay in G0/G1 transition	NIH3T3	[48]
Tctex-1 KD	↓	↓	G0/G1–S arrest	RPE1, 3T3, MEF	[49]
	(Non-ciliated cell lines)		Only marginal effects	HeLa, COS7	[49]
				IFT-20KD-RPE1	
				IFT88 mutant MEF	
Tctex-1 KD + Tctex-1 WT OE		→	Only marginal effects	3T3	[49]
Tctex-1 KD + Tctex-1 WT T99E OE		→	Only marginal effects	3T3	[49]
Tctex-1 KD + Tctex-1 WT T99A OE		↑	G0/G1–S arrest	3T3	[49]

*KD* knockdown, *OE* overexpression, *Rab8a Q67L* the constitutively active variant of Rab8a, *MEF* mouse embryonic fibroblast

^a^Inoko A., et al., unpubl. obs.

Sung and colleagues [49] reported new function of Tctex-1, a protein originally described as a light-chain subunit of cytoplasmic dynein [76, 77]. After serum stimulation in quiescent (ciliated) cells, Tctex-1 was phosphorylated at Thr94 and then targeted to the transition zone, the ciliary base between basal body and axoneme [49] (Fig. 2d). Tctex-1 depletion or replacement with its non-phosphorylated mutant suppressed ciliary absorption after serum stimulation, which in turn induced cell cycle arrest at the G0/G1–S transition [49] (Table 3). Conversely, replacement with its phospho-mimic mutant accelerated ciliary disassembly and entry into S phase after serum stimulation [49] (Table 3). The ciliary resorption via phosphorylated Tctex-1 was dependent on the actin cytoskeleton but independent of the Tctex-1 role of the cytoplasmic dynein components [49] (Fig. 2d). The cell cycle arrest by Tctex-1 depletion was seen in RPE1, 3T3, or MEF cell lines possessing the ability to form primary cilia during the quiescent state (G0/G1 phase) but not in HeLa and COS7 cell lines reducing this tendency [49] (Table 3). The cell cycle arrest in Tctex-1-depleted cells was reverted by the loss-of-function mutation of IFT88 or the co-depletion of IFT20 [49] (Table 3), the treatment to promote ciliary disassembly [28]. The authors also confirmed in vivo Tctex-1-Thr94-phosphorylation-dependent functions in radial glial cells; Tctex-1 depletion reduced the population of neural progenitor cells through premature differentiation of cortical neurons, whereas ectopic expression of the phospho-mimic mutant increased it [49].

Most recently, we found that Aurora-A activation by trichoplein (discussed in the former sections) is critical for the suppression of aberrant primary cilia formation during cell proliferation [47]. Trichoplein was concentrated at both centrioles in dividing cells [55], whereas it disappeared specifically from the basal body (which a mother centriole is converted to) in quiescent RPE1 cells [47]. Overexpressing trichoplein suppressed ciliary assembly in these quiescent cells [47]. Similarly, the microinjection of pre-activated Aurora-A was reported to induce ciliary disassembly in quiescent cells [44]. Conversely, in proliferating RPE1 cells, trichoplein or Aurora-A knockdown induced primary cilia formation, which resulted in cell cycle arrest at the G0/G1 phase [47]. This cell arrest reverted if primary cilia formation was blocked by simultaneously depleting IFT20 [47] or IFT88 (Inoko A., et al., unpubl. obs.) or by simultaneously treating with chloral hydrate [78] (Inoko A., et al., unpubl. obs.; Table 3). Unlike the RPE1 cell line, the silencing of trichoplein or Aurora-A failed to induce ciliogenesis or cell cycle arrest in the HeLa cell line where primary cilia are rarely formed in response to serum starvation [47] (Table 3).

These independent studies highlight the possibility that the presence of primary cilia can negatively influence cell cycle progression. The data on Nde1 [48] and Tctex-1 [49] propose a model that ciliary disassembly after cell cycle re-entry, per se, affects cell cycle progression especially at the G0/G1–S transition [74]. Our data are consistent with this model, but we can also suggest a broader model in which proper cell cycle progression requires continuous suppression of primary cilia formation in proliferating cells [47]. All these data clearly show that forced induction/suppression of primary cilia can affect cell cycle progression, in particular the transition from G0/G1 to the S phase.

In spite of the above recent publications [47–49], it is difficult to completely solve whether the absence of a primary cilium is a prerequisite for cell cycle progression because many treatments to influence ciliary dynamics are also known to have extra-ciliary effects [36, 37]. For example, IFT80 knockdown was reported to promote cell cycle progression to S and G2/M phases in (non-ciliated) HeLa cells [79]. IFT80 is also known to regulate mitotic spindle orientation [80]. IFT20 is localized not only in primary cilia but also in the Golgi apparatus [81]. The disruption of the actin cytoskeleton by cytochalasin D is known to activate several signaling pathways, such as the Rho signaling pathway [82]. However, these recent publications clearly show negative control results in their systems [47–49]. For example, IFT88 or IFT20 knockdown alone induced only marginal changes in cell cycle profile [47–49] (Table 3). Using several experimental conditions summarized in Table 3, these studies [47–49] independently show the inverse relationship between primary cilia and cell cycle progression, which reduces the risk of extra-ciliary (side) effect(s) in treatments to influence ciliary dynamics.

## Possible signaling to progress cell cycle in response to the absence of a primary cilium

Here, we discuss and speculate how the absence of a primary cilium leads to cell cycle progression. At present, it remains an unsolved question but several studies provide some clues. One possible pathway is to inhibit p53 function during cell cycle progression. Doxsey and colleagues [83] reported that p38 is activated by the depletion of several centrosome-associated proteins, some of which are implicated in primary cilia assembly. Then, p38 phosphorylates p53 at Ser33, which induces the expression of p21, one of the p53 target genes [83]. P21 inhibits cyclin/Cdk complex including cyclin A/Cdk2, which is required for S phase progression [83]. Interestingly, Aurora-A was reported to phosphorylate p53 at Ser215 and Ser315 and result in p53 inactivation [84, 85] (Table 2). The above observations suggest a possible role of p53 in monitoring centrosome integrity before the S phase, although there is no clear evidence about the relationship between the p53 pathway and primary cilia.

**Fig. 3:**
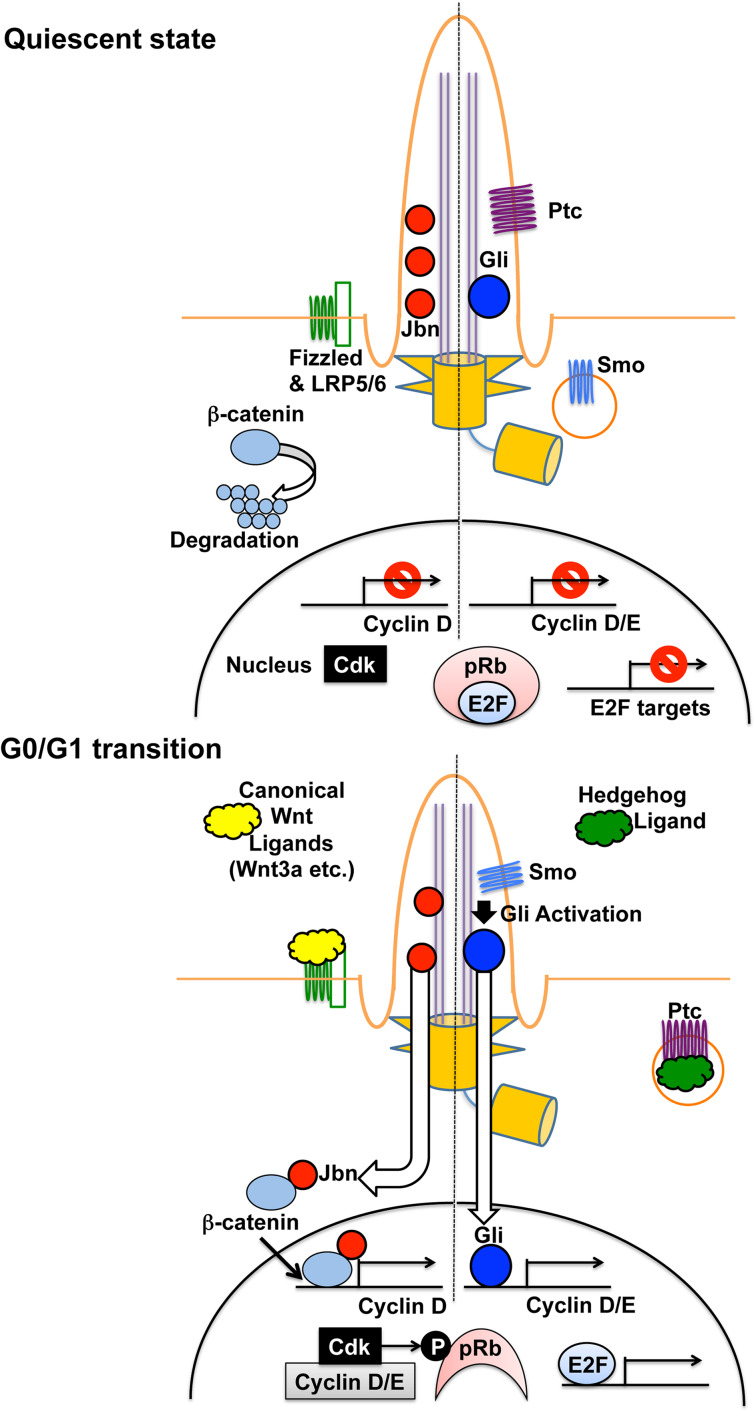
Effects of cilia-mediated signaling on cell cycle progression. In quiescent state (without the canonical Wnt ligands such as Wnt3a), β-catenin leads to degradation through the APC/GSK3β/Axin complex and Jouberin (Jbn) is sequestered in the cilium (*left*). Upon canonical Wnt ligand stimulation (at the G0/G1 transition), Jbn is translocated from the cilium to the cytoplasm and associated with β-catenin accumulating in the cytoplasm. The interaction between Jbn and β-catenin in the cytoplasm promotes the translocation of this complex to the nucleus, which in turn stimulates β-catenin-mediated transcription of target genes, such as cyclin D [91, 92]. On the other hand, in the absence of Hedgehog (Hh) ligand (in quiescent state), the Hh receptor Patched (Ptc) is localized in the primary cilium, where it suppresses the ciliary localization of Smoothened (Smo; *right*). Gli transcription factors are localized in the cilium but remain inactive. Upon Ptc association with Hh ligand (at the G0/G1 transition), Ptc is translocated to the cell body, which promotes Smo accumulation at the cilium. Smo activates Gli in the cilium, which accelerates the transcription of Gli target genes, such as cyclin D/E [35, 37, 86, 87, 89]. The expression of cyclin D/E leads to pRb hyperphosphorylation by Cdks associated with these cyclins. This hyperphosphorylation induces E2F-mediated gene expression, which advances cell cycle from G1 to S phase [16–18]

The other pathway is to elevate the expression of G1 cyclins (D and E types), which contribute to cell cycle transition from G1 to S through pRb phosphorylation [16–18] (summarized in Fig. 3). It appears to be controlled by two putative signaling pathways, Shh and canonical Wnt. Shh is a soluble ligand for Patched (Ptc), the transmembrane receptor localized in a primary cilium in its inactive state [35, 37, 86, 87]; loss of cilia failed to respond to Shh [88]. Ptc represses the activity of Smoothened (Smo) in the absence of Shh. Upon Ptc stimulation by Shh, Ptc moves out of a cilium and relieves Smo inhibition [35, 37, 86, 87]. Conversely, Smo enters the cilium and then stimulates the Gli family of transcription factors, which activates several genes such as cyclins D and E [35, 37, 86, 87, 89]. On the other hand, the relationship between primary cilia and Wnt signaling pathways is still under debate [35, 37, 90]. Gleeson and colleagues demonstrated an important role of a primary cilium in the inhibition of canonical Wnt pathway [91, 92]. In quiescent cells, Jouberin (Jbn), a protein associated with Joubert syndrome (a classic ciliopathy), was sequestered in cilia but was released from the cilia upon the stimulation of the canonical Wnt pathway [91, 92]. Jbn then interacts with β-catenin, which facilitates the activation of β-catenin-mediated transcription of target genes, such as cyclin D [91, 92]. The expression of cyclin D/E leads to pRb hyperphosphorylation by Cdks associated with these cyclins. This hyperphosphorylation induces E2F-mediated gene expression, which progresses cell cycle from G1 to S phase [16–18]. In support of these observations, Tctex-1 depletion, which blocked ciliary resorption, reduced pRb phosphorylation and resulted in cell cycle arrest at the G0/G1–S transition [49].

## Signaling pathways upstream of Aurora-A

We here consider how Aurora-A may be activated by the growth factor-mediated signaling, in particular through calcium signaling. Tucker and colleagues first observed loss of cilia in cells treated with platelet-derived growth factor (PDGF) or calcium ionophores [34]. Increase in free intracellular calcium is one of earliest events stimulated by growth factors including PDGF [35, 93–95] (Fig. 1). Recently, Golemis and colleagues [96] reported that Aurora-A was activated in response to the elevation of intracellular calcium. This activation was mediated by Aurora-A binding to calcium–calmodulin (Ca^2+^/CaM) [96] and required for Aurora-A-dependent ciliary disassembly [46] (Fig. 1; Table 1). However, this Ca^2+^/CaM-mediated activation was relatively short (within 3 min) in contrast to Aurora-A activation observed in ciliary resorption [46, 96]. Since Ca^2+^/CaM also enhanced the binding between Aurora-A and HEF1 [46], Aurora-A activity may be maintained by HEF1 (likely by Pifo) after the reduction of free intracellular calcium (Fig. 1).

With regard to HEF1 stability during ciliary disassembly, Lee and colleagues pointed out the importance of the non-canonical Wnt pathway [97]. Wnt5a-mediated ligand stimulation triggered casein kinase 1 epsilon (CK1ε) activation, which induced Dishevelled 2 (Dvl2) phosphorylation at Ser143 and Ser224 [97]. This phosphorylation created docking sites for Plk1 [97], one of the mitotic kinases [40, 43, 98–100]. Dvl2-bound Plk1 inhibited Smad3-dependent HEF1 degradation [97]. This Wnt5a–CK1ε–Dvl2/Plk1-mediated HEF1 stabilization enhanced ciliary resorption by Aurora-A [97, 101]. These findings support a close relationship between primary cilia and Wnt signaling pathways, but it is still being debated [35, 37, 90].

Tctex-1 function in ciliary resorption also depends on the Aurora-A/HEF1 complex [49] (Fig. 2). In a separate study, the expressions of both Aurora-A and HEF1 were elevated by mutation-induced loss of a protein associated with von Hippel–Lindau disease [102]. Thus, upstream molecules regulating Aurora-A/HEF1-mediated deciliation start to emerge, but less information is available about the mechanism of the molecular switch among Aurora-A activators, such as Ca^2+^/CaM, HEF1, Pifo, and trichoplein during cell proliferation.

## Conclusions and perspectives

The primary cilium is a dynamic organelle whose assembly and disassembly appear to be linked to cell cycle. A series of recent publications have strongly suggested the possibility that the absence of a primary cilium may be a prerequisite for cell cycle progression especially at the G0/G1–S transition [47–49]. Due to technical limitations and some exceptions, this possibility remains in dispute. However, the argument is fed by observations that most cancer cells lack cilia [103–108] and that ciliary formation is suppressed by Aurora-A [44–47], a putative oncogene [109, 110]. Interestingly, the inhibition of Aurora-A can cause fatal mitotic errors in tumor cells, whereas it may induce healthy cells to merely assemble cilia and exit the cell cycle [47]. This difference of cellular reaction may potentially make Aurora-A an attractive target for anti-cancer therapies.

Recent studies also raise a new question of why forced induction/absorption of a primary cilium affects cell cycle progression. One possible explanation is the preparation of centrosome duplication in the S phase: a basal body and its associated daughter centriole may not serve as templates for centriole duplication. On the other hand, many ciliated protozoans are known to duplicate their centrioles without deciliation [111]. Therefore, whether entry into the S phase requires the absence of a primary cilium may depend on cells in which deciliated centrioles are a prerequisite for duplication templates. Further investigation will be needed to clarify the role of primary cilia (dis)assembly on cell cycle progression.
